# Spatiotemporal change of cultivated land in China during 2000–2020

**DOI:** 10.1371/journal.pone.0293082

**Published:** 2024-01-17

**Authors:** Wenqi Zhang, Ruiqing Qie

**Affiliations:** School of Economics and Management, Jilin Agricultural University, Changchun, China; Policy Research Institute, NEPAL

## Abstract

Cultivated land is of great significance for ensuring food security. Since the 21st century, China’s rapid development has led to urban construction occupying a lot of cultivated land. The understanding of stage characteristics and regional differences needs to be improved. And it is necessary to quantify the temporal and spatial pattern of cultivated land changes since the 21st century. We used the global land cover dataset (GlobeLand30) to investigate the quantity and spatial distribution of cultivated land change in China from 2000 to 2020. The results show that: 1) Over the last two decades, China’s arable land has diminished by 54,996 square kilometers. Notably, the arable land loss between 2010 and 2020 was 1.74 times greater than that from 2000 to 2010. This decline has been exacerbated by factors such as the expansion of urban and rural areas, as well as initiatives to revert farmland to forests and grasslands; 2) The eastern coastal regions experienced the most severe net arable land loss, with a net reduction of 42,989 square kilometers from 2000 to 2020, primarily driven by urban expansion; 3) In contrast, the western regions demonstrated the most substantial net increase in arable land, expanding by 11,583 square kilometers from 2000 to 2020, primarily driven by the development of forests and grasslands. It is noteworthy that despite some areas successfully implementing policies to return farmland to forests and grasslands, the ecologically fragile western regions continue to experience accelerated conversion of these natural landscapes into arable land, partly to compensate for the farmland decrease driven by urbanization in the eastern regions. Looking ahead, China’s cultivated protection policy must strike a balance between the ecological value of the western region and the economic value of the eastern region.

## 1. Introduction

China is the most populous country on Earth. China has been facing a serious food security problem [1–3]. With the continuous development of China’s economy and the improvement of people’s living standards, the demand for food, biofuels and other commodities is also growing, which poses a great challenge to China’s cultivated land area and intensive degree [4–7]. Cultivated land is the foundation for supporting food production and maintaining sustainable agricultural development, and the study of spatiotemporal changes in cultivated land is crucial for formulating scientific and reasonable land use management policies. Since the 21st century, China has entered an unprecedented process of Land-Use and Land-Cover Change. Driven by China’s macro policies, rapid urbanization and industrial expansion have led to a large number of high-quality and high-yield cultivated land being swallowed up. It has also brought about drastic changes in the spatial pattern of land use. With the high-quality development of China’s society, the central and local governments urgently need to master the area, distribution and change information of the cultivated land for solving major problems such as ecology, resources, environment and agricultural production [8–10]. At the same time, China implements the strictest cultivated land protection measures in the world. It will increase the area of cultivated land to a certain extent [11–13]. The United Nations 2030 sustainable development goals (SDGs) emphasize the need to increase agricultural production while ensuring ecological security [14]. Therefore, the continuous monitoring of the change in cultivated land area and scope in China is of great significance to the realization of SDGs and the optimization and adjustment of China’s agricultural production policies.

At present, there are three main methods to obtain the temporal and spatial information of cultivated land change: (1) Based on the level-by-level data submitted by national and local statistical departments, most of them take administrative units as the basic units, with long time series and highly comprehensive data, which is difficult to reflect the spatial differences within administrative units. (2) National land survey. Since the 1980s, China has carried out three national land surveys, which are the basic basis for major national conditions and national strength surveys and the formulation of major strategic policies. However, the time is fixed and discontinuous, and the detailed spatial data are not publicly available, which is difficult to meet the needs of macro level research. (3) Remote sensing monitoring, this method has strong timeliness, high spatial resolution, low cost and wide audience. Remote sensing technology plays a crucial role in the study of land use change. It can provide comprehensive and high-resolution surface information, effectively monitor and analyze spatiotemporal changes in land use, and help to deeply understand the spatial distribution and influencing factors of land use. In addition, remote sensing technology can also provide historical data, enabling researchers to compare land use changes over different periods, thereby revealing long-term trends and changes. Therefore, it is very important to obtain land use change information in different time periods based on remote sensing time and analyze the change trend of China’s cultivated land to ensure food security and protect the ecological environment.

In order to obtain the spatial information of cultivated land change in time, scholars began to use remote sensing technology to monitor the relevant information of cultivated land change for decision-making, protection and restoration. From 1980 to 2000, more than 55% of new agricultural land was at the cost of destroying intact forests [15]. The farmland in South America has increased by 160% since 1985, which has seriously affected the ecological service function of the region [16]. The soybean planting area in South America more than doubled from 2000 to 2019, and 9% of the forest loss in the whole South American continent was transformed into soybean [17]. The degree of global cultivated land intensification exceeded the degree of expansion by analyzing the GlobeLand30 dataset from 2000 to 2010 [18]. Many studies have shown that cultivated land is expanding at the global level, which affects the ecosystem balance in many areas.

Using the publicly released high-precision long-time series land use data to monitor the change of China’s cultivated land is of great strategic significance for answering the major issues of ecology, resources, environment and agricultural production that the state is concerned about, formulating relevant macro policies, and meeting the national and regional sustainable development needs. The GlobeLand30 dataset is the first global geographic information public product provided by China to the United Nations. It has been praised by international peer experts as "a milestone in the open sharing of earth observation and geographic information". The GlobeLand30 dataset has been widely used in land cover change analysis [19–21] and has been proven to have higher accuracy than similar products [22–24]. Compared to self analysis data, we choose to use GlobeLand30 data for land use change analysis, which has higher authority and reliability. The GlobeLand30 data provides land use classification data for the years 2000, 2010, and 2020, covering the land use situation throughout China, facilitating the study of spatiotemporal changes [25, 26]. In addition, the GlobeLand30 data adopts a consistent classification system and standards, making data from different periods comparable and conducive to revealing the characteristics and trends of spatiotemporal changes in cultivated land [27].

For this purpose, we use the global geographic information public product GlobeLand30 to evaluate the change of cultivated land in China since the beginning of the 21st century. We specifically aim (1) to describe the area of cultivated land change since the 21st century, including the transformation of cultivated land into other land types and the transformation of other land types into cultivated land; (2) to explore the spatial and temporal pattern of cultivated land change in different regions of China; and (3) to discuss the impact of cultivated land trends on China’s food security in the future.

## 2. Materials and methods

### 2.1 Mapping cultivated land areas in China

GlobeLand30 is the world’s first global land cover dataset with a resolution of 30 m [25]. The classified images used in the development of GlobeLand30 data are mainly 30 m multispectral images, including the TM5, ETM +, and OLI multispectral images of Landsat and the multispectral images of HJ-1. The 2020 version of the data also uses 16 m resolution and high resolution multispectral images (GF-1) [25]. GlobeLand30 dataset include 10 primary types: cultivated land, forest, grassland, shrubland, wetland, water body, tundra, artificial surfaces, bareland, permanent snow and ice [28]. We mainly involve cultivated land, artificial surface, forest, grassland and water body, among which cultivated land refers to lands used for agriculture, horticulture and gardens, and artificial surface refers to lands modified by human activities, and forest refers to lands covered with trees, with vegetation cover over 30%, and grassland refers to lands covered by natural grass with cover over 10%, and water body refers to water bodies in the land area. The overall accuracy of the GlobeLand30 V2010 dataset is 83.50%, and the Kappa coefficient is 0.78. The overall accuracy of the GlobeLand30 V2020 dataset is 85.72%, and the Kappa coefficient is 0.82 [29]. Previous studies have shown that the overall accuracy of GlobeLand30 in China is 81.0%, which can meet the needs of this study [30–33]. For more detailed information about GlobeLand30, please refer to http://www.globallandcover.com. GlobeLand30 has high spatial resolution and high classification accuracy, which makes it possible to analyze land use change on a national scale. We used three datasets, namely, the datasets of 2000, 2010 and 2020, through ArcGIS 10.2 to extract a spatial distribution map of cultivated land in China.

### 2.2 Temporal and spatial analysis of cultivated land change

The study divides the 21st century into two stages, with 2000–2010 as the Stage 1 and 2010–2020 as the Stages 2. The time interval of the two stages is consistent and the data meet the research needs. The total change in cultivated land area in a certain period is limited, but the transfer in and out between cultivated land and other land types is very frequent. We use ArcGIS 10.2 software for quantitative analysis of cultivated land change and transfer characteristics, statistics of the specific destination of cultivated land loss in different stages and the specific source of expanded cultivated land. The pattern and process of cultivated land use are synthesized and extracted through the Geoscience Information Atlas.

**Fig 1:**
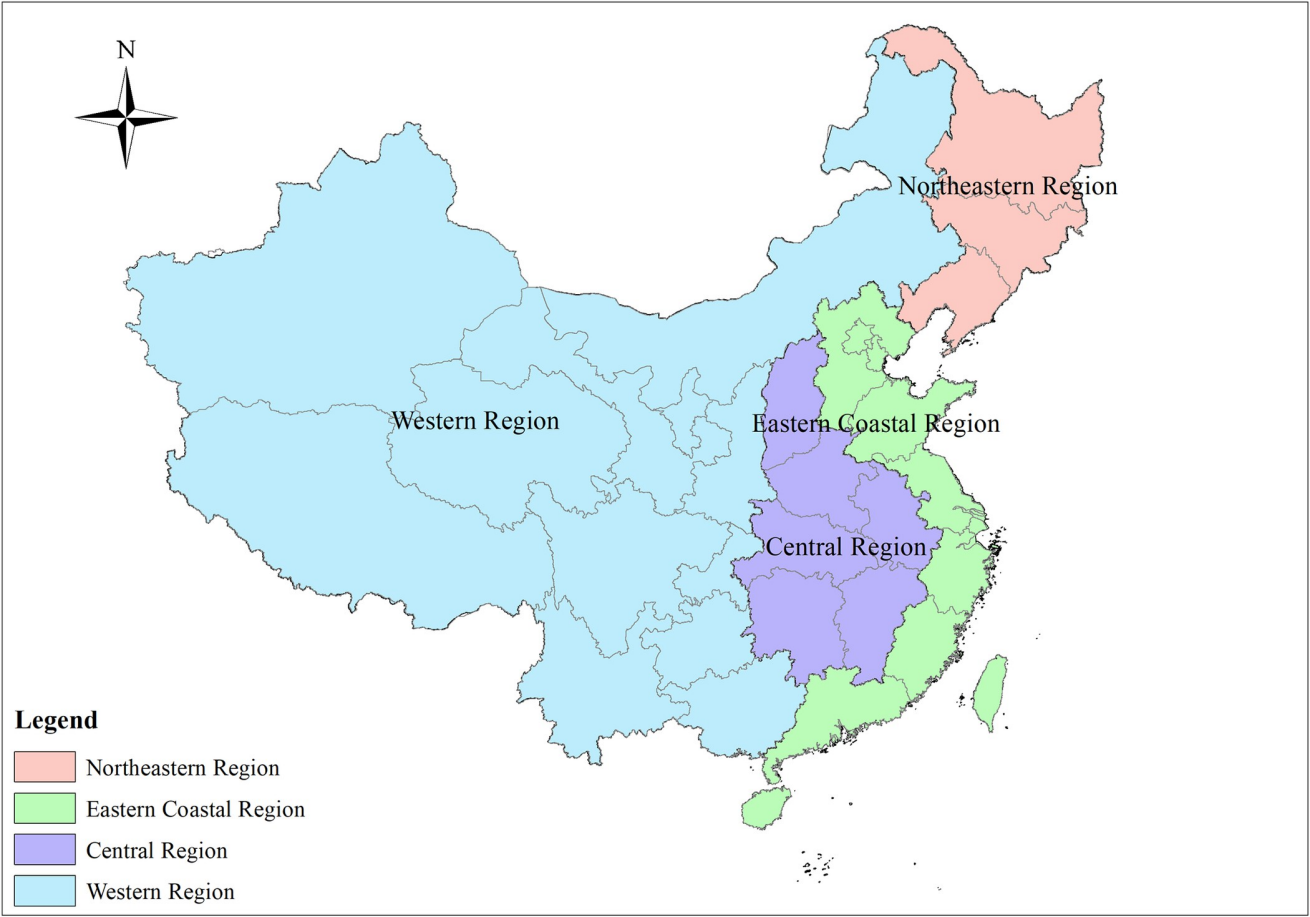
Regional division of China. Note: This drawing has not been previously copyrighted. The authors created the image themselves.

To better reflect the regional characteristics and regional differences in cultivated land change, we divide China into four regions (Fig 1): the northeastern region (NER), eastern coastal region (ECR), central region (CR) and western region (WR) [34]. The natural environment, economic development and regional characteristics of each region are different, and the increase and decrease in cultivated land are different [35].

## 3. Results

### 3.1 Quantitative characteristics of cultivated land change in China from 2000 to 2020

Through the statistical analysis of cultivated land data extracted by GlobeLand30, it can be seen that the total area of cultivated land in China has decreased since the 21st century. In 2000, China’s cultivated land area was 210.5×10^4^ km^2^, in 2010, 208.5×10^4^ km^2^, and in 2020, 205×10^4^ km^2^. In the Stage 1, China’s cultivated land area decreased by 20000 km^2^, and in the Stage 2, China’s cultivated land area decreased by 35000 km^2^. Due to the expansion of artificial land in urban and rural areas, ecological restoration, agricultural reclamation and other reasons, the cultivated land changes more frequently and the cultivated land area decreases faster. In the Stage 2, compared with the Stage 1, the loss (Table 1) and the expansion (Table 2) of cultivated land in China are rising in value. Since the beginning of the 21st century, the loss of cultivated land in China has mainly shifted to artificial surfaces, forests, grassland and water bodies, which account for more than 97% of the total loss of cultivated land (Table 1).

**Table 1:** Changing area of cultivated land loss in China during 2000–2020 (km^2^).

Types	2000–2010		2010–2020	
	Area	Ratio	Area	Ratio
Cultivated land → forest	51877	31.47%	77610	30.04%
Cultivated land → grassland	51251	31.09%	53391	20.66%
Cultivated land → shrubland	2104	1.28%	2214	0.86%
Cultivated land → wetland	1594	0.97%	1982	0.77%
Cultivated land → water body	13753	8.34%	18194	7.04%
Cultivated land → artificial surfaces	42810	25.97%	102926	39.83%
Cultivated land → bareland	1427	0.87%	2050	0.79%

**Table 2:** Changing area of cultivated land expansion in China during 2000–2020 (km^2^).

Types	2000–2010		2010–2020	
	Area	Ratio	Area	Ratio
Forest →cultivated land	55328	38.21%	73203	32.76%
Grassland →cultivated land	46848	32.36%	92830	41.55%
Shrubland →cultivated land	1759	1.22%	4259	1.91%
Wetland →cultivated land	2616	1.81%	2931	1.31%
Water body →cultivated land	13072	9.03%	14042	6.28%
Artificial surfaces →cultivated land	20368	14.07%	21588	9.66%
Bareland →cultivated land	4788	3.31%	14577	6.52%

In the Stage 1, the area of cultivated land converted into forest was the largest, accounting for 31.47% of the total loss area of cultivated land. The area of cultivated land converted into grassland was the second largest, accounting for 31.09% of the total loss area of cultivated land. At the same time, 25.97% of the total loss area of cultivated land was converted to artificial surfaces, and 8.34% of the total loss area of cultivated land was converted to water body, and a small amount was converted to other land types. It shows that in this stage, affected by the policy of returning cultivated land to forest and grassland, many cultivated land has been converted to ecological land, and a certain proportion of cultivated land has also been converted to artificial surfaces.

In the Stage 2, the area of cultivated land lost was changed into artificial surfaces at most, accounting for 39.83% of the total cultivated land lost. The area of lost cultivated land converted into forest is only second to artificial surfaces, accounting for 30.04% of the total loss area of cultivated land at this stage. At the same time, 20.66% of the total loss area of cultivated land is converted to forest, 7.04% of the total loss area of cultivated land was converted to water body, and a small amount was converted to other land types. This shows that in the second stage, the area of cultivated land developed as artificial surface is increasing.

Since the 21st century, the cultivated land expanded in China mainly comes from grassland and forest, accounting for more than 70% of the total area of cultivated land expansion in this stage, followed by artificial surface and water body (Table 2).

In the Stage 1, the proportion of expanded cultivated land from forest to cultivated land was the highest, 38.12%. Secondly, the area from grassland to cultivated land accounted for 32.63% of the total expanded cultivated land area. In addition, some of the expanded cultivated land has changed from artificial surfaces, water body and other land use types, accounting for a relatively small proportion.

In the Stage 2, the expanded cultivated land came from grassland, forest, artificial surfaces and bareland in order of area. The area from grassland to cultivated land accounted for the largest proportion of expanded cultivated land area, 41.55%. At the same time, 32.76% of the expanded cultivated land area was transformed from forest, followed by artificial surface and bare land. At the same time, 32.76% of the expanded cultivated land area was transformed from forest, followed by artificial surfaces and bare land.

### 3.2 Spatial characteristics of cultivated land change from 2000 to 2020

**Fig 2:**
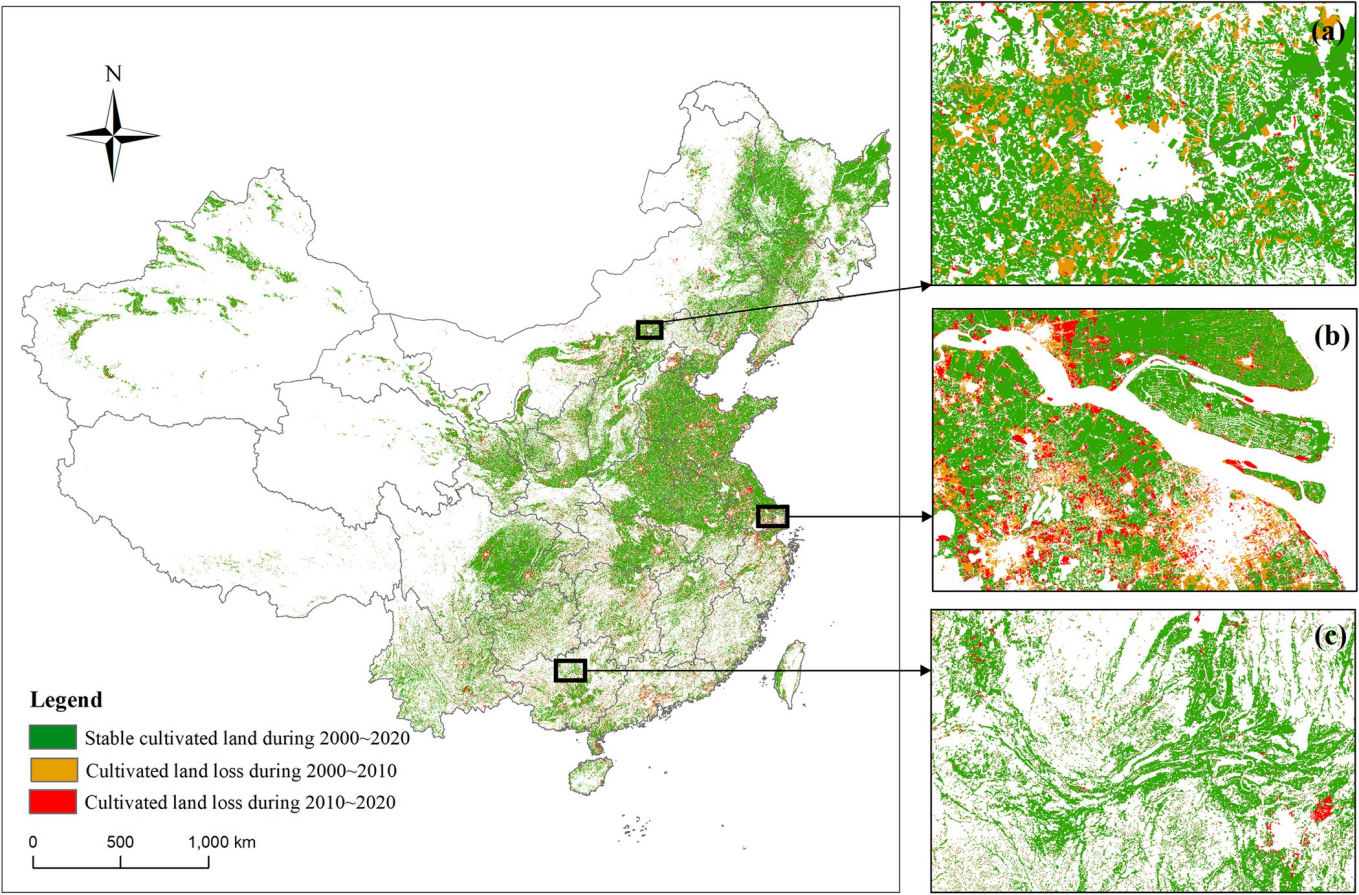
Spatial characteristics of cultivated land loss in China during 2000~2020. Note: This drawing has not been previously copyrighted. The authors created the image themselves.

The spatial pattern, loss status and loss characteristics of typical regions of cultivated land in China are shown in Fig 2. The cultivated land is mainly distributed on the Northeast Plain, North China Plain, middle and lower reaches of the Yangtze River Plain and other plains or low hilly areas, while the cultivated land in the west is less and scattered. From 2000 to 2020, about 90% of China’s cultivated land was stable, that is, the land use types in 2000, 2010 and 2020 were all cultivated land. In the two stages, 2000–2010 and 2010–2020, the conversion of cultivated land to forest in the north (Fig 2A), urban expansion in the middle-east (Fig 2B) and nonagricultural cultivated land in the southwest (Fig 2C) caused serious damage to cultivated land.

**Fig 3:**
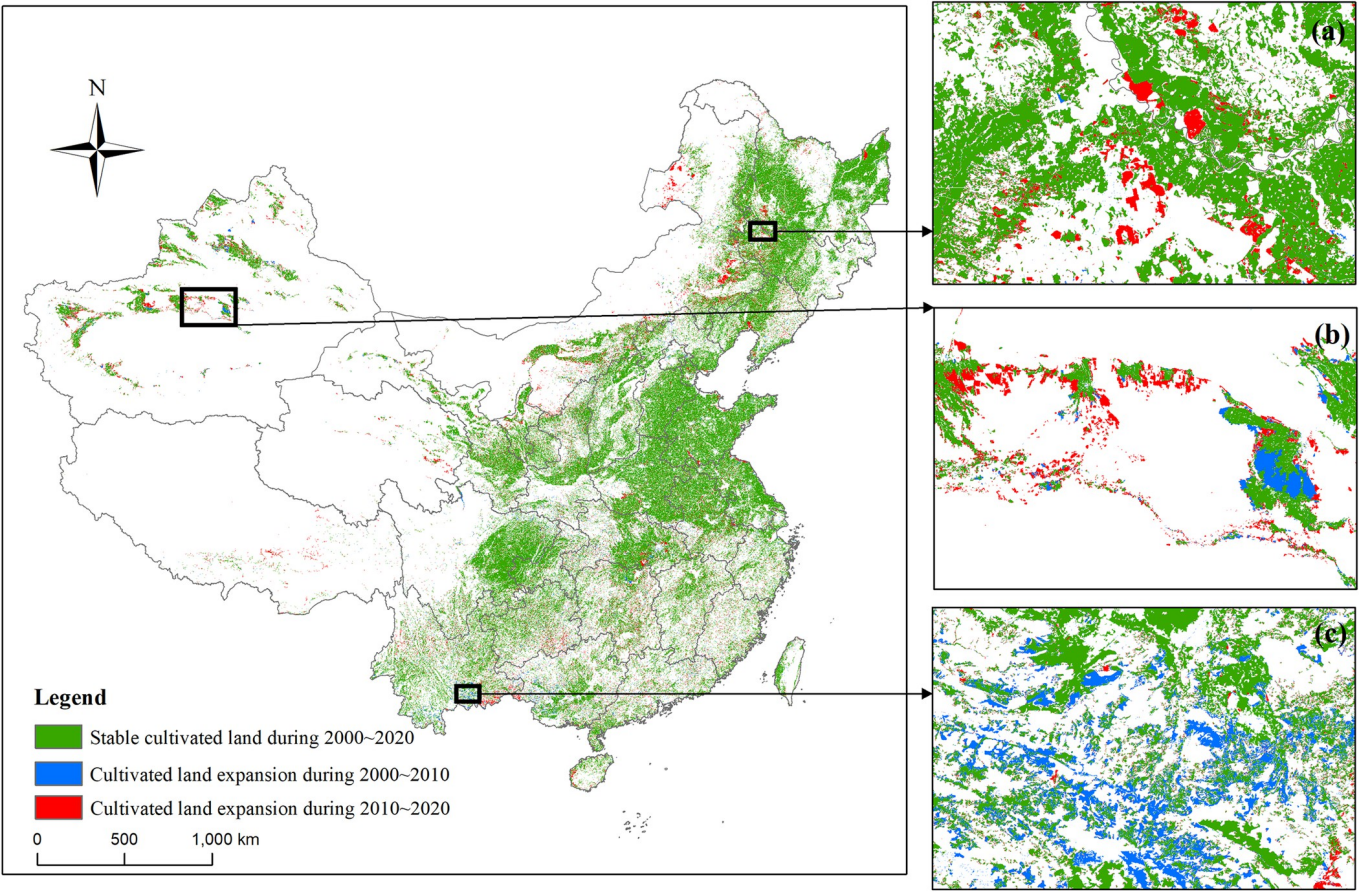
Spatial characteristics of cultivated land expansion in China during 2000~2020. Note: This drawing has not been previously copyrighted. The authors created the image themselves.

The spatial pattern, expansion status and expansion characteristics of typical regions of cultivated land in China are shown in Fig 3. Since the 21st century, the expanded cultivated land is mainly distributed in the NER and WR. In the Stage 1, 144800 km^2^ of cultivated land was added, and in the Stage 2, 223400 km^2^ of cultivated land was added. The speed of cultivated land expansion was accelerating. Driven by economic interests, some forest, grassland and saline alkali land have been reclaimed as cultivated land. In addition, Many studies have shown that China’s cultivated land dynamic balance policy has a significant positive effect on cultivated land area [36–38]. As a result, the expansion of cultivated land in recent years comes from the reclamation of saline alkali wasteland in Northeast China (Fig 3A), the reclamation of agricultural wasteland in Northwest China (Fig 3B) and the land consolidation and development in the Southwest China (Fig 3C).

### 3.3 Regional characteristics of cultivated land change from 2000 to 2020

The sum of the increase and decrease of the cultivated land area can reflect the net change of the total cultivated land area in the region (Table 3). Since the 21st century, the changing rate of cultivated land in China’s four regions has been increasing, among which the loss of cultivated land in the NER, ECR and CR has accelerated, and the expansion of cultivated land in the WR has accelerated. In the Stage 1, the total area of cultivated land in NER decreased by 1662 km^2^, the total area of cultivated land in ECR decreased by 14648 km^2^, the total area of cultivated land in CR decreased by 4696 km^2^, and the total area of cultivated land in WR increased by 967 km^2^. In the Stage 2, the total area of cultivated land in NER decreased by 5032 km^2^, the total area of cultivated land in ECR decreased by 28341 km^2^, the total area of cultivated land in CR decreased by 12199 km^2^, and the total area of cultivated land in WR increased by 10616 km^2^.

**Table 3:** Net change of total cultivated land area in various regions of China since the 21st century (km^2^).

Stage	NER	ECR	CR	WR	TOTAL
2000–2010	-1662	-14648	-4696	967	-20040
2010–2020	-5032	-28341	-12199	10616	-34956

**Fig 4:**
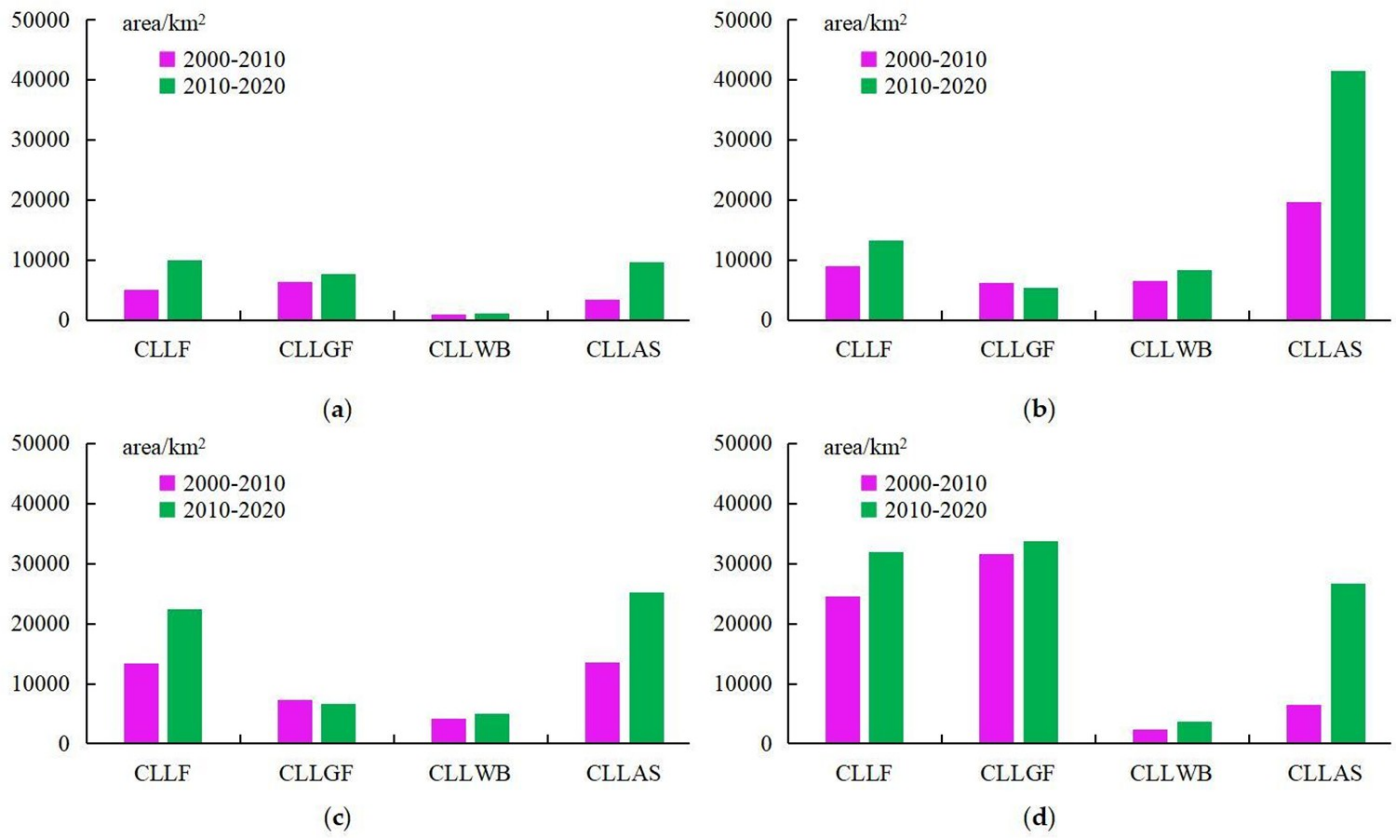
Spatial variations in cultivated land loss induced by the expansion of different types at the scale of geographic regions in China: Figure a-d represents four regions of China. CLLF denotes cultivated land loss due to the expansion of forest; CLLGL denotes cultivated land loss due to the expansion of grassland; CLLWB denotes cultivated land loss due to the expansion of water bodies; and CLLAS denotes cultivated land loss due to the expansion of artificial surfaces.

Fig 4 records the changes in cultivated land loss in different regions. Compared with the four regions in China, the amount of cultivated land loss in NER is the smallest (Fig 4A). Compared with the four regions in China, the loss of cultivated land caused by returning cultivated land to forests is relatively large in CR(Fig 4C) and WR(Fig 4D) of China. Among them, in the Stage 1, the area of cultivated land lost to forest in CR and WR is 13330 km^2^ and 24583 km^2^ respectively. In the Stage 2, the area of cultivated land lost to forest in CR and WR is 22400 km^2^ and 31981 km^2^ respectively. The area of cultivated land converted to forest in the Stage 2 is higher than that in the Stage 1. Returning cultivated land to grassland is particularly prominent in the WR(Fig 4D), and the area of cultivated land converted to grassland is significantly higher than that of other regions. In the two stages, the area of cultivated land converted to grassland in the WR is 31525 km^2^ and 33754 km^2^ respectively. The main reason for the loss of cultivated land in the ECR is urban expansion (Fig 4B), especially in the Stage 2.

**Fig 5:**
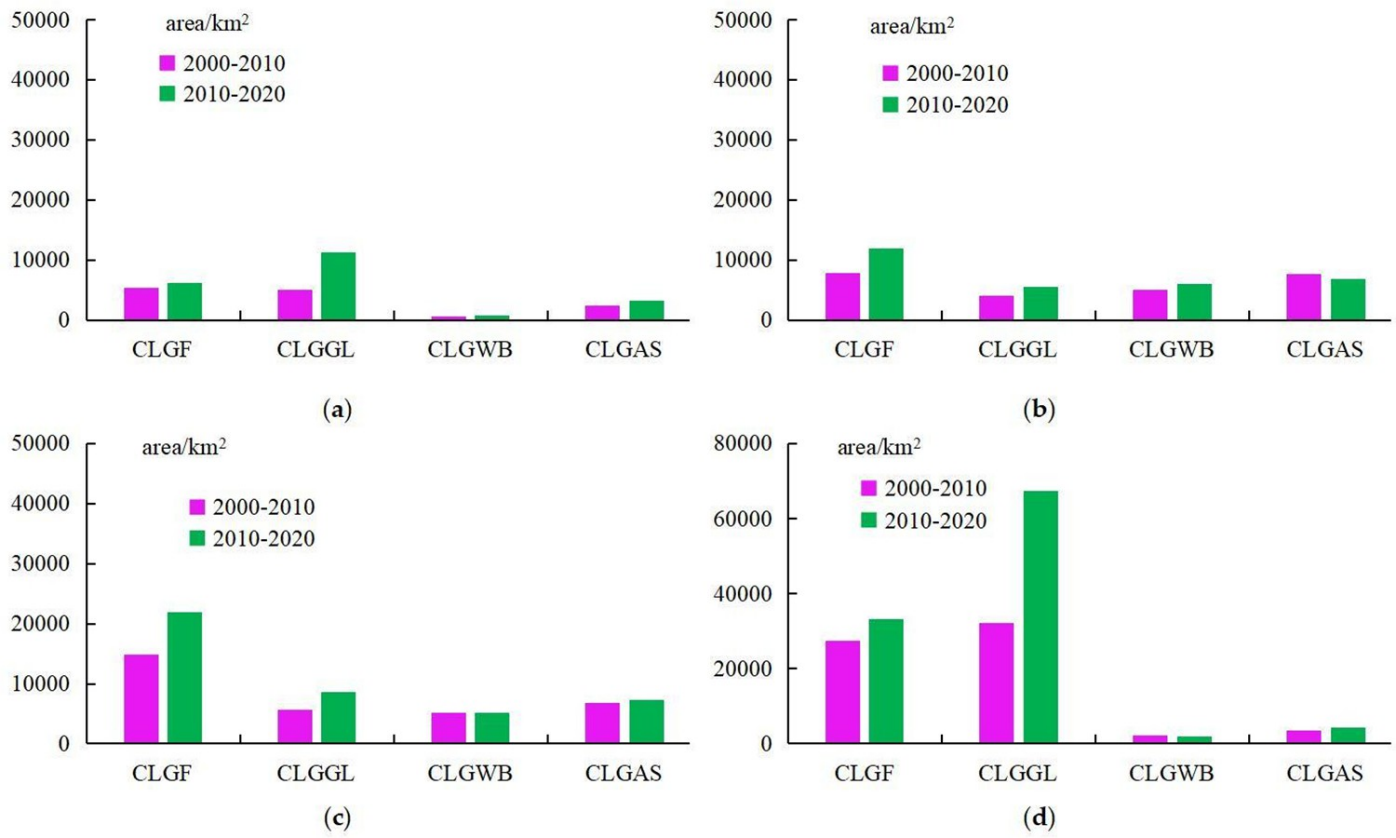
Spatial variations in cultivated land expansion induced by the loss of different types at the scale of geographic regions in China: Figure a-d represents four regions of China. CLGF denotes cultivated land gain from forest loss; CLLGL denotes cultivated land gain from grassland loss; CLLWB denotes cultivated land gain from water body loss; and CLLAS denotes cultivated land gain from artificial surface loss.

Fig 5 records the changes in cultivated land expansion in different regions. Compared with the four regions in China, the area of cultivated land expansion in the WR is the largest (Fig 5D). In the two stages, 64861 km^2^ and 96165 km^2^ of cultivated land were expanded respectively, and the phenomenon of grassland consolidation into cultivated land in the Stage 2 is particularly prominent. Forest is the main source of cultivated land expansion in the ECR (Fig 5B) and the CR (Fig 5C). In the Stage 1, the area of forest converted to cultivated land in the CR and the WR is 7767 km^2^ and 14772 km^2^ respectively. In the Stage 2, the area of forest converted to cultivated land in the CR and the WR is 11853 km^2^ and 21916 km^2^ respectively. At the same time, in the ECR and the CR, there is a phenomenon of reclaiming lakes for cultivated land, and part of the water body is changed into cultivated land. The area of forest and grassland reclaimed into cultivated land in the NER is more than that of water body and artificial surface converted into cultivated land(Fig 4A). The area of forest and grassland converted into cultivated land in the Stage 1 is 5324 km^2^ and 4951 km^2^ respectively, and the area of forest and grassland converted into cultivated land in the Stage 2 is 6153 km^2^ and 11254 km^2^ respectively.

## 4. Discussion

### 4.1 Spatial and temporal changes of cultivated land in China based on the Globeland30

The aim of this study is to utilize Globeland30 data to analyze the spatiotemporal changes of cultivated land in China from 2000 to 2020, and reveal its regional characteristics. Compared with field surveys, using remote sensing images to identify the areas where cultivated land increases and decreases is more rapid and efficient, especially at the national scale [39–41]. Our study uses the mature and accurate land cover product GlobeLand30 to analyze the temporal and spatial pattern of cultivated land change in China within four regions since 2000. Previous relevant studies have usually only analyzed the temporal and spatial changes in China’s cultivated land from 1990 to 2000 or 2000 to 2010 [42, 43]. Compared with these studies, we used the latest GlobeLand30 dataset from 2020 and analyzed the changes in China’s cultivated land from 2010 to 2020 for the first time. The period from 2000 to 2020 is the fastest urbanization stage in China’s history [44, 45], and the period when China’s cultivated land protection policies and legislation tended to be improved [46, 47]. In conclusion, extracting the spatiotemporal changes of cultivated land in China based on Globeland30 data is an effective method that provides strong support for in-depth understanding of the evolution of Chinese farmland. Through this analysis, we can obtain comprehensive and detailed information about the changes in Chinese cultivated land, which can serve as a scientific basis for land management and decision-making. Our analytical results have greater guiding significance for the existing policies.

### 4.2 Main reasons for the loss and expansion of cultivated land in China

According to the transfer characteristics of land use types in the eastern coastal region, the artificial surface accounted for the largest proportion of cultivated land loss from 2000 to 2020. At the same time, from the perspective of space, the conversion of cultivated land to artificial surface mostly occurred in the surrounding areas of cities. Therefore, it is inferred that urbanization is the first factor in the reduction of cultivated land. In addition, returning cultivated land to forest and grassland in the western region is also one of the factors leading to the reduction in cultivated land. However, compared with almost irreversible urbanization, the transformation of cultivated land into forest and grassland in the western region will improve ecosystem service functions in the region [48, 49]. In particular, the returned farmland areas are usually slope cultivated land and desertification and salinization cultivated land with low yield and serious soil and water loss. The cultivated land occupied by urbanization in the eastern coastal region is usually high-quality cultivated land with strong cultivated land production capacity [50]. This will undoubtedly have a large impact on grain production, and relevant studies have also shown that the grain production capacity in the eastern coastal region is declining [51, 52]. In addition, to supplement the cultivated land occupied by urbanization, some grassland and forest have been reclaimed as cultivated land, which will undoubtedly affect the security of the local ecological environment.

From the perspective of time, the conversion of cultivated land into grassland and forest from 2000 to 2010 was the main factor leading to the reduction in cultivated land, which is similar to the results of previous studies using statistical data [43]. Compared with 2000–2010, the conversion of cultivated land into artificial surfaces also became the main factor for the reduction in cultivated land in 2010–2020. From 2010 to 2020, the urbanization process of the whole country accelerated. In 2010–2020, the area of cultivated land converted to artificial surfaces in the four regions of the country generally exceeded twice the area of cultivated land converted to artificial surfaces in 2000–2020, especially five times in the western region. The acceleration of China’s urbanization in 2010–2020 eventually led to the accelerated reduction of cultivated land in 2010–2020 compared with 2000–2010. China’s urbanization rate reached 63.89% in 2020. China’s urbanization has entered a period of steady development, and the number of cultivated land occupied by urbanization will decrease in the next 10 years. Researchers utilized the 30-meter annual land cover dataset developed by Yang and Huang [53] in China, and their findings aligned with the conclusions drawn in this study, thereby validating the reliability of our research [54].

### 4.3 Deficiencies and prospects

This study conducted an in-depth analysis of the spatiotemporal changes of cultivated land in China using the Globeland30 dataset. Although China was divided into four regions based on geographical differences, and the spatiotemporal characteristics of land loss and land expansion were analyzed, there are still some limitations to this study.

One limitation is that the temporal resolution of the Globeland30 dataset is 10 years, which may restrict our ability to make fine-grained observations of changes in Chinese cultivated land. Land use changes can occur at shorter time scales, and higher temporal resolution data would better reflect these changes. Therefore, future research could explore the use of higher-resolution remote sensing datasets to provide more accurate and detailed spatiotemporal information on changes in cultivated land.

China is a vast country with complex geography. This study divided China into four major regions for comparative analysis, but this coarse-grained regional division may not capture finer regional differences. Land use conditions in different regions of China may exhibit significant variations. Therefore, future research could consider a more detailed regional division to obtain more accurate regional characteristics and patterns of cultivated land changes.

It is worth noting that China’s large-scale urbanization has consumed a large amount of cultivated land. Our research shows that although China has implemented the most stringent cultivated land protection system. However, due to the impact of urbanization, more cultivated land was converted into artificial surfaces in 2010–2020 than in 2000–2010, and the reduction rate of cultivated land accelerated instead of decreasing. We suggest that Chinese policy makers should pay more attention to the accelerated loss of cultivated land and further optimize protection measures to reduce the rate of cultivated land loss. In addition, urbanization has led many farmers to give up agriculture and work in cities [55], and the resulting social contradictions also need to be considered, especially in the eastern coastal region and western region of China, which have recently experienced rapid urbanization. In view of the requirements for the orderly development of land space and the harmonious development among regions for the construction of "beautiful China" in the new era of China. We must formulate scientific policies and regulations, implement and enforce them to minimize the disappearance of cultivated land, strengthen the protection of cultivated land, and more reasonably manage and intensively use the remaining cultivated land.

## 5. Conclusions

According to the cultivated land change information from 2000 to 2020 extracted from the global land cover product Globeland30, we determined the quantity and spatial distribution of cultivated land change in China. The research shows that the rate of cultivated land reduction in China has an obvious increasing trend and significant spatial differences. From 2010 to 2020, the net loss of cultivated land in China was 34956 km^2^, which was significantly faster than the net loss of 20400 km^2^ from 2000 to 2010. In the Stage 1 and the Stage 2, 164800 km^2^ and 258400 km^2^ of cultivated land were transformed into other land types in China, most of which occurred in the eastern coastal region and western regions. The eastern coastal region mainly flows to artificial surface, and the western part mainly flows to grassland. At the same time, 144700 km^2^ and 223400 km^2^ of other land types were converted into cultivated land in the Stage 1 and Stage 2, respectively, most of which occurred in the western and central regions. China has made great efforts in the protection of cultivated land, but since the 21st century, the acceleration of China’s urbanization has led to the accelerated loss of cultivated land. As China enters a new era, in order to take the quality of development and the construction of ecological civilization as the fundamental goals, we must more strictly implement the cultivated land protection system to prevent more cultivated land from disappearing and affecting the national food security.
